# SD-VIS: A Fast and Accurate Semi-Direct Monocular Visual-Inertial Simultaneous Localization and Mapping (SLAM)

**DOI:** 10.3390/s20051511

**Published:** 2020-03-09

**Authors:** Quanpan Liu, Zhengjie Wang, Huan Wang

**Affiliations:** School of Mechatronical Engineering, Beijing Institute of Technology, Beijing 100081, China; liuquanpan@126.com (Q.L.); WH978992767@163.com (H.W.)

**Keywords:** visual-inertial, semi-direct SLAM, multi-sensor fusion

## Abstract

In practical applications, how to achieve a perfect balance between high accuracy and computational efficiency can be the main challenge for simultaneous localization and mapping (SLAM). To solve this challenge, we propose SD-VIS, a novel fast and accurate semi-direct visual-inertial SLAM framework, which can estimate camera motion and structure of surrounding sparse scenes. In the initialization procedure, we align the pre-integrated IMU measurements and visual images and calibrate out the metric scale, initial velocity, gravity vector, and gyroscope bias by using multiple view geometry (MVG) theory based on the feature-based method. At the front-end, keyframes are tracked by feature-based method and used for back-end optimization and loop closure detection, while non-keyframes are utilized for fast-tracking by direct method. This strategy makes the system not only have the better real-time performance of direct method, but also have high accuracy and loop closing detection ability based on feature-based method. At the back-end, we propose a sliding window-based tightly-coupled optimization framework, which can get more accurate state estimation by minimizing the visual and IMU measurement errors. In order to limit the computational complexity, we adopt the marginalization strategy to fix the number of keyframes in the sliding window. Experimental evaluation on EuRoC dataset demonstrates the feasibility and superior real-time performance of SD-VIS. Compared with state-of-the-art SLAM systems, we can achieve a better balance between accuracy and speed.

## 1. Introduction

Simultaneous localization and mapping (SLAM) plays an important role in self-driving cars, virtual reality, unmanned aerial vehicles (UAV), augmented reality and artificial intelligence [1,2]. This technology can provide reliable state estimation for UAV and self-driving cars in GPS-denied environments by relying on its sensors. Various types of sensors can be utilized in SLAM, such as stereo camera, lidar, inertial measurement units (IMU), and monocular camera. However, they have significant disadvantages when used individually: the metric scale of stereo camera can be obtained directly by using fixed baseline length, but it can only be estimated accurately in a limited depth range [3]; lidar has high precision in indoor, but it will encounter the reflection problem of glass surface in outdoor [4]; cheap IMUs are extremely susceptible to bias and noise [5]; monocular camera cannot estimate the absolute metric scale [6]. This paper mainly studies the monocular vision-inertial navigation system (VINS) based on multi-sensor fusion [7], which has the advantages of small size, lightweight, observable scale, roll, and pitch angle, etc.

According to the different methods of image information processing, there are two categories of SLAM: feature-based method and direct method. The standard process of feature-based method proceeds in three steps [8,9,10]. Firstly, a group of sparse point or line features is extracted from each image, and feature matching between adjacent frames is performed by using invariant feature descriptors. Secondly, estimate the camera motion and the 3D position of sparse feature points by using multiple view geometry theory. Finally, optimize the camera motion and the 3D position of sparse feature points by minimizing visual re-projection errors. However, the feature-based method has the following disadvantages: feature extraction and matching are very time-consuming; fewer features extractable in low-texture environments; repeated texture environments will cause incorrect feature matching. Therefore, the accuracy and robustness of the feature-based method depend on the correct feature extraction and matching.

The direct method considers the entire image or some pixels with a large gradient and directly estimates the camera motion and scene structure by minimizing the photometric error [11,12,13,14]. Therefore, in the low-texture environments and repeated texture environments, the performance of the direct method is better than the feature-based method. In addition, without feature extraction and matching, the calculation speed of the direct method is faster than the feature-based method. However, the photometric error function is highly non-convex, it is difficult for the direct method to converge when large baseline motion and image blur occurs. Due to the inability to perform effective loop closure detection, the cumulative drift generated during long-term operation is still an unresolved problem [15].

Academic research on SLAM is very extensive. Well known methods include PTAM, SVO, VINS-Mono, LSD-SLAM, ORB-SLAM, DSO. PTAM was one of the most representative systems in the early stage of the feature-based SLAM algorithm. This algorithm first proposed to divide tracking and mapping into two parallel threads [16]. After that, most feature-based SLAM algorithms have adopted this idea, including ORB-SLAM. ORB-SLAM is the most successful feature-based SLAM, which uses ORB features in tracking, mapping, re-location, and loop closure detection [17]. VINS- Mono is a compelling multi-sensor monocular vision-inertial SLAM based on tight coupling and non-linear optimization [18]. Pl-VIO is an extension of the line feature of VINS-Mono, which can optimize the re-projection error of point and line features in a sliding window [19]. VINS-Fusion is a multi-sensor fusion platform based on non-linear optimization, which is the continuation of VINS-Mono in the direction of multi-sensor fusion [20]. LSD-SLAM is a novel method of real-time monocular SLAM based on direct method and can create large-scale semi-dense maps in real-time on a laptop without GPU acceleration [21]. Based on LSD-SLAM, DSO adds complete photometric calibration and uniformly samples pixels with large gradients throughout the image, thereby improving tracking accuracy and robustness [11]. On the basis of DSO, LDSO adds loop closure detection, which makes up for the shortcomings of DSO incapable of loop closure detection and eliminates the cumulative error generated during long navigation [22]. Stereo DSO is an improved version of DSO, which can realize the real-time estimation of motion and 3D structure with high accuracy and robustness on the moving stereo camera [3]. Lee authors in [7] presents a new implementation method for efficient simultaneous localization and mapping using a forward-viewing monocular vision sensor. The method is developed to be applicable in real time on a low-cost embedded system for indoor service robots.

In recent years, SLAM systems that combine the feature-based method and direct method have become popular. Semi-direct visual odometry (SVO) is an effective hybrid method that combines the advantages of feature-based method and direct method [23]. However, since the algorithm was originally designed for the drone’s look-down camera, it is easy to lose camera tracking in other settings. Lee [24] proposed a loose-coupled method by combining ORB-SLAM and DSO to improve positioning accuracy. However, its frontend and backend are almost independent, which cannot share estimation information to further improve the pose precision. In [25,26], different semi-direct approaches were proposed for stereo odometry. Both methods use feature-based tracking to obtain a motion prior, and then perform direct semi-dense or sparse alignment to refine the camera pose. SVL [27] can be considered a combination of ORB-SLAM and SVO. The method for ORB-SLAM is adopted in keyframes, and SVO is adopted in non-keyframes. Therefore SVL can achieve good balance between speed and accuracy according to camera motion and environment. Our method is motivated by SVL, but we go one step further in real-time performance. Specifically, thanks to the keyframe selection strategy and sliding window-based back-end, we only need to extract new feature points on the keyframes and track them with KLT sparse optical flow algorithm, which can further reduce the calculation complexity while ensuring accuracy.

In this paper, we present SD-VIS, a novel fast and accurate semi-direct visual-inertial SLAM framework, which combines the exactness of feature-based method and quickness of direct method. The keyframes in SD-VIS are tracked by feature-based method, which is used for sliding window-based non-linear optimization and loop closure detection. This strategy solves the problem of drift in the long-term operation and ensures the robustness of the algorithm in case of large baseline motion and image blur. Non-keyframes are tracked by the direct method, and the distance between adjacent non-keyframes is minimal, which ensures the convergence of error function. Compared with the direct method, SD-VIS exhibits the function of loop closure detection and solves the problem of drift in long-term operation. Compared with the feature-based method, SD-VIS can achieve the same accuracy while maintaining a faster speed. 

## 2. System Framework Overview

**Figure 1:**
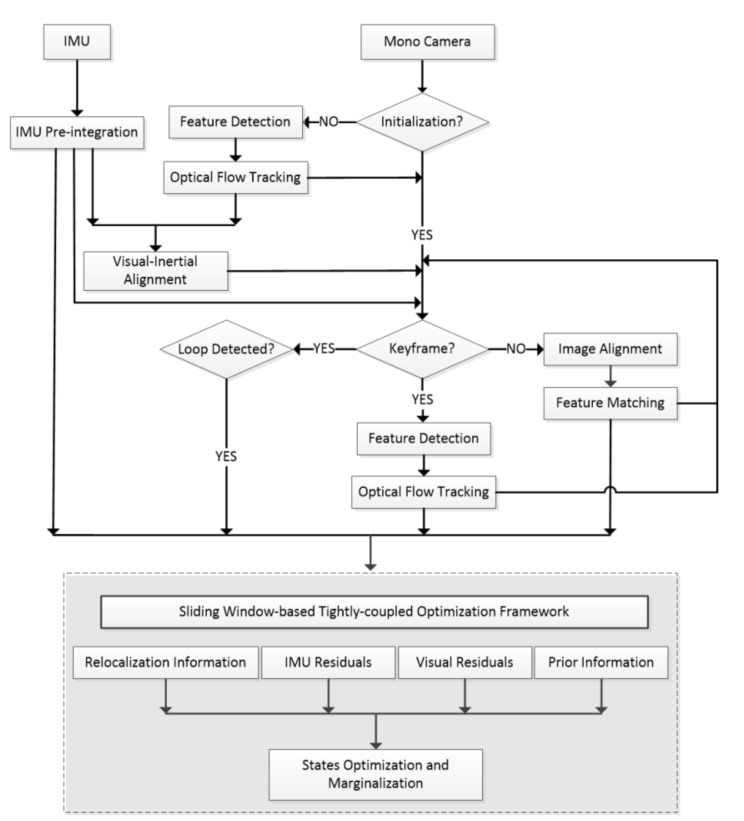
The semi-direct visual-inertial SLAM system framework

Figure 1 demonstrates the framework of the semi-direct vision-inertial SLAM system. Sensor data comes from a monocular camera and IMU. IMU measurements between two consecutive images are pre-integrated, and the pre-integration is used as the constraint of IMU between two images (Section 3.2). In the initialization procedure, we detect the feature points on each image and track them with KLT sparse optical flow algorithm [15].

In the following visual-inertial alignment, we align the pre-integrated IMU measurements and visual images and calibrate out the metric scale, initial velocity, gravity vector, and gyroscope bias by using multiple view geometry (MVG) theory based on the feature-based method. (Section 3.3). After initialization, keyframe selection will be performed based on the IMU pre-integration and previous feature matching results. The previous feature matching refers to the feature matching between the last frame and the penultimate frame in the sliding window, and the matching is completed before the current frame arrives. Non-keyframes are used for fast-tracking and localization by direct method [11], and keyframes are tracked by feature-based method [18] and used for non-linear optimization and loop closure detection (Section 4). In the following tight coupling optimization framework, we can get more accurate state estimation by minimizing visual re-projection error, IMU residual, prior information from marginalization, and re-location information from loop closure detection (Section 5).

## 3. IMU Measurements and Visual-Inertial Alignment

### 3.1. Definition of Symbols

**Figure 2:**
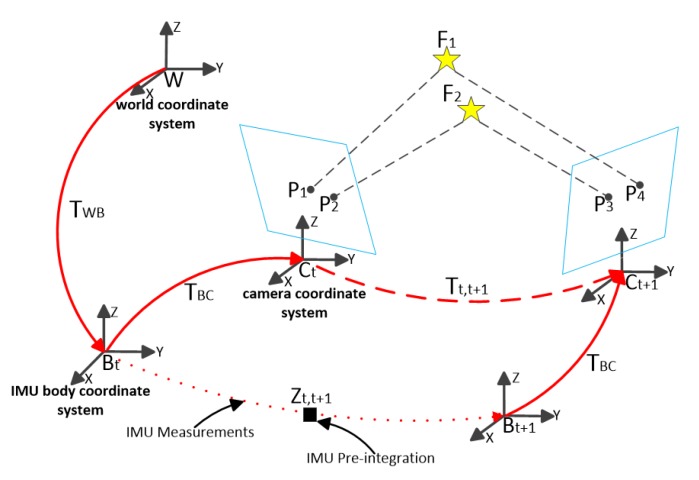
Symbol definition of algorithm

Figure 2 shows the definition of symbols in the semi-direct visual-inertial SLAM framework. C, B, and W are the camera coordinate system, the IMU body coordinate system, and the world coordinate system, respectively. We define $T_{\mathrm{WB}}=\left(R_{B}^{W},P_{B}^{W}\right)\;$ as the motion of B relative to W. $T_{\mathrm{BC}}=\left(R_{C}^{B},P_{C}^{B}\right)\;$ represents the extrinsic parameters between C and B, which can be calibrated in advance. $T_{t,t+1}\in \;\mathrm{SE}\left(3\right)$ represents the motion from time t to time t+1 in the coordinate system C, and $Z_{t,t+1}$ represents a pre-integrated IMU measurement between the camera coordinate system $C_{t}$ and $C_{t+1}$.

A 3D point $F_{1},{\;F}_{2}\in R^{3}$ represents the spatial feature points observed simultaneously by the camera coordinate system $C_{t}\;$ and $C_{t+1}$. $P_{1},{\;P}_{2},{\;P}_{3},P_{4}\in R^{2}$ are the projections of feature points on the image coordinate system. We adopt the traditional pinhole camera model to map the $F_{1}$ in the camera coordinate system to the image coordinate system by the projection function π: $R^{3}\to R^{2}$:

(1) $$P_{1}={\pi F}_{1}=\left[\begin{array}{c} f_{u}\frac{x_{c}}{z_{c}}+c_{u}\\ f_{v}\frac{y_{c}}{z_{c}}+c_{v} \end{array}\right]F_{1}={\left[\begin{array}{ccc} x_{c} & y_{c} & z_{c} \end{array}\right]}^{T}$$

where ${\left[\begin{array}{cc} f_{u} & f_{v} \end{array}\right]}^{T}$ and ${\left[\begin{array}{cc} c_{u} & c_{v} \end{array}\right]}^{T}$ is camera internal parameters.

### 3.2. IMU Pre-Integration

In the back-end optimization and visual-inertial alignment, the constraints of vision and IMU need to be optimized in the same frame, so the IMU measurements between two adjacent frames need to be integrated into one constraint.

IMU can output 3-axis angular velocity $\tilde{\omega }$ and 3-axis acceleration $\tilde{\alpha }$ including bias and Gaussian white noise:

(2) $$\tilde{\omega }=\omega^{B}+b_{\omega }^{B}+n_{\omega }^{B}$$

(3) $$\tilde{\alpha }=R_{W}^{B}\left(\alpha^{W}+g^{W}\right)+b_{\alpha }^{B}+n_{\alpha }^{B}$$

where $n_{\omega }^{B}\;\sim N\left(0,\sigma_{\omega }^{2}\right)$, $n_{\alpha }^{B}\;\sim N\left(0,\sigma_{\alpha }^{2}\right)$ represents the Gaussian white noise. ${\;g}^{W}={\left[0,0,\;g\right]}^{T}$ is the gravity vector. $R_{w}^{B}$ represents the rotation matrix from W to B. $b_{\omega }^{B},{\;b}_{\alpha }^{B}$ represents the biases of gyroscope and accelerometer.

We define $P_{B_{i}}^{W}$,${\;V}_{B_{i}}^{W}$, $q_{B_{i}}^{W}$ as the translation, velocity, and rotation quaternions in the IMU body coordinate system $B_{i}$. If we know the measurements value of IMU during t∈[i,j], we can calculate $P_{B_{j}}^{W}$, $V_{B_{j}}^{W}$, $q_{B_{j}}^{W}$ in the coordinate system $B_{j}$:

(4) $$P_{B_{j}}^{W}=P_{B_{i}}^{W}+V_{B_{i}}^{W}\Delta t+\iint_{t\in \left[i,j\right]}^{\;}\left[R_{B_{t}}^{W}\left({\tilde{\alpha }}_{t}-b_{\alpha_{t}}^{B}\right)-g^{W}\right]{\delta t}^{2}$$

(5) $$V_{B_{j}}^{W}=V_{B_{i}}^{W}+\int_{t\in \left[i,j\right]}^{\;}\left[R_{B_{t}}^{W}\left({\tilde{\alpha }}_{t}-b_{\alpha_{t}}^{B}\right)-g^{W}\right]\delta t$$

(6) $$q_{B_{j}}^{W}=q_{B_{i}}^{W}⊗\int_{t\in \left[i,j\right]}^{\;}\frac{1}{2}\Omega \left({\tilde{\omega }}_{t}-b_{\omega_{t}}^{B}\right)q_{B_{t}}^{W}\delta t$$

where:

$$\Omega \left(\omega \right)=\left[\begin{array}{cc} -\omega_{\times } & \omega \\ -\omega^{T} & 0 \end{array}\right],\;\omega_{\times }=\left[\begin{array}{ccc} 0 & -\omega_{z} & \omega_{y}\\ \omega_{z} & 0 & -\omega_{x}\\ -\omega_{y} & \omega_{x} & 0 \end{array}\right]$$

From formulas (4)–(6), we can know that the IMU body state $P_{B_{j}}^{W}$, $V_{B_{j}}^{W}$, $q_{B_{j}}^{W}$ is related to the IMU body coordinate system $B_{i}$. In the back-end tightly coupling optimization, we will continuously iteratively update the IMU state variables in the sliding window. When the IMU body state at time t=i is iteratively updated, we need to recalculate the state at time t=j, which is very time-consuming. We adopt IMU pre-integration technology to avoid unnecessary time consumption. formulas (4)–(6) can be written as:

(7) $$R_{W}^{B_{i}}P_{B_{j}}^{W}=R_{W}^{B_{i}}\left(P_{B_{i}}^{W}+V_{B_{i}}^{W}\Delta t-\frac{1}{2}g^{W}{\Delta t}^{2}\right)+\theta_{B_{j}}^{B_{i}}$$

(8) $$R_{W}^{B_{i}}V_{B_{j}}^{W}=R_{W}^{B_{i}}\left(V_{B_{i}}^{W}\Delta t-g^{W}{\Delta t}^{\;}\right)+\beta_{B_{j}}^{B_{i}}$$

(9) $$q_{W}^{B_{i}}⊗q_{B_{j}}^{W}=\gamma_{B_{j}}^{B_{i}}$$

where:

(10) $$\theta_{B_{j}}^{B_{i}}=\iint_{t\in \left[i,j\right]}^{\;}\left[R_{B_{t}}^{B_{i}}\left({\tilde{\alpha }}_{t}-b_{\alpha_{t}}^{B}\right)\right]{\delta t}^{2}$$

(11) $$\beta_{B_{j}}^{B_{i}}=\int_{t\in \left[i,j\right]}^{\;}\left[R_{B_{t}}^{B_{i}}\left({\tilde{\alpha }}_{t}-b_{\alpha_{t}}^{B}\right)\right]\delta t$$

(12) $$\gamma_{B_{j}}^{B_{i}}=\int_{t\in \left[i,j\right]}^{\;}\frac{1}{2}\Omega \left({\tilde{\omega }}_{t}-b_{\omega_{t}}^{B}\right)q_{B_{t}}^{B_{i}}\delta t$$

From formulas (10)–(12), the pre-integration measurements $\theta_{B_{j}}^{B_{i}}$, $\beta_{B_{j}}^{B_{i}}$, $\gamma_{B_{j}}^{B_{i}}$, and the IMU body coordinate system $B_{i}$ are independent of each other. This means that when the states in the $B_{i}$ coordinate system are iteratively updated, there is no need to recalculate the states in the $B_{j}$ coordinate system. Since the IMU pre-integrated measurements $\theta_{B_{j}}^{B_{i}}$, $\beta_{B_{j}}^{B_{i}}$, $\gamma_{B_{j}}^{B_{i}}$ is affected by the bias, when the bias is updated iteratively, we will update the pre-integrated measurement by the first-order approximation method:

(13) $$\theta_{B_{j}}^{B_{i}}\approx {\tilde{\theta }}_{B_{j}}^{B_{i}}+J_{b_{\alpha }}^{\theta }{\delta b}_{\alpha_{\;}}^{B}+J_{b_{\omega }}^{\theta }{\delta b}_{\omega_{\;}}^{B}$$

(14) $$\beta_{B_{j}}^{B_{i}}\approx {\tilde{\beta }}_{B_{j}}^{B_{i}}+J_{b_{\alpha }}^{\beta }{\delta b}_{\alpha_{\;}}^{B}+J_{b_{\omega }}^{\beta }{\delta b}_{\omega_{\;}}^{B}$$

(15) $$\gamma_{B_{j}}^{B_{i}}\approx {\tilde{\gamma }}_{B_{j}}^{B_{i}}⊗\left[\begin{array}{c} 0\\ \frac{1}{2}J_{b_{\omega }}^{\gamma }{\delta b}_{\omega_{\;}}^{B}{}_{\;} \end{array}\right]$$

where $J_{b_{\alpha }}^{\theta }$, $J_{b_{\omega }}^{\theta }$, $J_{b_{\alpha }}^{\beta }$, $J_{b_{\omega }}^{\beta }$, $J_{b_{\omega }}^{\gamma }$ are the Jacobian matrices of pre-integrated measurements with respect to bias.

### 3.3. Visual-Inertial Alignment

The convergence speed and effect of nonlinear visual-inertial SLAM systems depend heavily on reliable initial values. Therefore, in the initialization procedure of SD-VIS, we align the pre-integrated IMU measurements with the visual image to complete the system initialization.

#### 3.3.1. Gyroscope Bias Correction

We regard the camera coordinate system $C_{0}$ as the world coordinate system. We detect the feature points on each image and track them with KLT sparse optical flow algorithm, and then the rotation $R_{C_{t}}^{C_{0}}$ and $R_{C_{t+1}}^{C_{0}}$ of the two adjacent frames $C_{t}$ and $C_{t+1}$ can be estimated by using visual structure from motion (SfM). Rotation increment ${\tilde{\gamma }}_{B_{t+1}}^{B_{t}}$ between the IMU body coordinate system $B_{t}$ and $B_{t+1}$ can be estimated by IMU pre-integration. We can get the following formula:

(16)  δbωtBmin∑​‖RCt+1C0−1⊗RCtC0⊗γBt+1Bt‖2

where:

(17) $$\gamma_{B_{t+1}}^{B_{t}}\approx {\tilde{\gamma }}_{B_{t+1}}^{B_{t}}⊗\left[\begin{array}{c} 0\\ \frac{1}{2}J_{b_{\omega }}^{\gamma }{\delta b}_{\omega_{t}}^{B}{}_{\;} \end{array}\right]$$

We solve the above least squares problem to get the initial calibration of the gyroscope bias and use it to update $\theta_{B_{j}}^{B_{i}}$, $\beta_{B_{j}}^{B_{i}}$, $\gamma_{B_{j}}^{B_{i}}$.

#### 3.3.2. Gravity Vector, Initial Velocity, and Metric Scale Correction

We define the variables that need to be calibrated as:

(18) $$X_{I}=\left[\begin{array}{ccc} V_{b_{0}}^{b_{0}} & V_{b_{1}}^{b_{1}} & \begin{array}{cc} \ldots & \begin{array}{cc} V_{b_{n}}^{b_{n}} & g^{C_{0}} \end{array}\;s \end{array} \end{array}\right]$$

where $V_{b_{k}}^{b_{k}}$ is the initial velocity in the IMU body coordinate system,${\;g}^{C_{0}}$ is the gravity vector in the camera coordinate system, s represents the metric scale of semi-direct visual-inertial SLAM framework.

Suppose we have obtained pre-calibrated external parameter $T_{\mathrm{BC}}=\left(R_{C}^{B},P_{C}^{B}\right)$, we can transform the pose $T_{C_{0}C_{t}}=\left(R_{C_{t}}^{C_{0}},P_{C_{t}}^{C_{0}}\right)$ from the camera coordinate system to the IMU body coordinate system:

(19) $$q_{B_{t}}^{C_{0}{}_{\;}}=q_{C_{t}}^{C_{0}{}_{\;}}⊗q_{C}^{B}{}^{-1}$$

(20) $$sP_{B_{t}}^{C_{0}{}_{\;}}={\mathrm{sP}}_{C_{t}}^{C_{0}{}_{\;}}-R_{B_{t}}^{C_{0}{}_{\;}}P_{C}^{B}$$

Considering two adjacent keyframes $B_{t}$ and $B_{t+1}$, then formulas (7) and (8) can be rewritten as:

(21) $$\theta_{B_{t+1}}^{B_{t}}=R_{C_{0}}^{B_{t}}\left(s\left(P_{B_{t+1}}^{C_{0}}-P_{B_{t}}^{C_{0}}\right)-R_{B_{t}}^{C_{0}}V_{B_{t}}^{B_{t}}\Delta t+\frac{1}{2}g^{C_{0}}{\Delta t}^{2}\right)$$

(22) $$\beta_{B_{t+1}}^{B_{t}}=R_{C_{0}}^{B_{t}}\left(R_{B_{t+1}}^{C_{0}}V_{B_{t+1}}^{B_{t+1}}-R_{B_{t}}^{C_{0}}V_{B_{t}}^{B_{t}}+g^{C_{0}}{\Delta t}^{\;}\right)$$

We combine formulas (19)–(22) to get the following formula:

(23) $${\tilde{Z}}_{B_{t+1}}^{B_{t}}=\left[\begin{array}{c} \theta_{B_{t+1}}^{B_{t}}-P_{C}^{B}+R_{C_{0}}^{B_{t}}R_{B_{t+1}}^{C}P_{C}^{B}\\ \beta_{B_{t+1}}^{B_{t}} \end{array}\right]=H_{B_{t+1}}^{B_{t}}X_{I}+n_{B_{t+1}}^{B_{t}}$$

where:

(24) $$H_{B_{t+1}}^{B_{t}}=\left[\begin{array}{ccc} -I\Delta t & 0 & \begin{array}{cc} \frac{1}{2}R_{C}^{B_{t}}{\Delta t}^{2} & {\;R}_{C_{0}}^{B_{t}}\left(P_{C_{t+1}}^{C_{0}}-P_{C_{t}}^{C_{0}}\right) \end{array}\\ -I & R_{C_{0}}^{B_{t}}R_{B_{t+1}}^{C_{0}} & \begin{array}{cc} R_{C_{0}}^{B_{t}}\Delta t & \;\;\;\;\;\;0 \end{array} \end{array}\right]$$

In the above formula $R_{B_{t}}^{C_{0}}$, $R_{B_{t+1}}^{C_{0}}$,${\;P}_{B_{t}}^{C_{0}}$, $P_{B_{t+1}}^{C_{0}}$ can be obtained through visual SfM:

(25)  XI min∑​‖Z˜Bt+1Bt−HBt+1BtXI‖2

Solving the above formula, we can calibrate the initial velocity for each keyframe, gravity vector, and absolute metric scale. After estimating the scale, we will adjust the translation vector of the vision SfM to make the system have an observable scale.

#### 3.3.3. Gravity Vector Refinement

We can know the magnitude of the gravity vector in advance, so we refer to the VINS-Mono [18] method to re-parameterize the gravity vector obtained in Section 3.3.2 with two variables in tangent space, and perform further optimization.

After obtaining the accurate gravity vector, we can rotate the coordinate system $C_{0}$, which is temporarily the world coordinate system, to the real world coordinate system W. However, since the yaw angle in the visual-inertial SLAM system is unobservable, the yaw angle of the $C_{0}$ coordinate system remains unchanged during the rotation process. At this time, the initialization procedure of the semi-direct visual-inertial SLAM system is completed.

## 4. Visual Measurements

### 4.1. Keyframe Selection

We have three different keyframe selection strategies. Satisfying one of these three strategies makes the current frame a keyframe. The first and third strategies of keyframe selection are based on the feature matching results of the last frame and the penultimate frame in the sliding window, which has been matched before the current frame arrives. The first selection strategy is the tracking number of feature points. No new feature points will be extracted when tracking non-keyframes. The translational motion of the camera will lead to the decrease of tracking feature points. If the number of tracking points in the last frame in the sliding window is less than 70% of the minimum tracking point threshold, the current frame will be treated as a keyframe. The second selection strategy is related to IMU pre-integration. If the translation distance between the last two adjacent frames in the sliding window calculated by the IMU pre-integration exceeds a preset threshold, the current frame is also considered as a new keyframe. The third selection strategy is the average parallax of the feature points tracked on the the last frame and the penultimate frame in the sliding window. The translation or rotation of camera will cause parallax. When the average parallax exceeds the threshold, the current frame will also be regarded as a keyframe.

### 4.2. Keyframes Tracking

**Figure 3:**
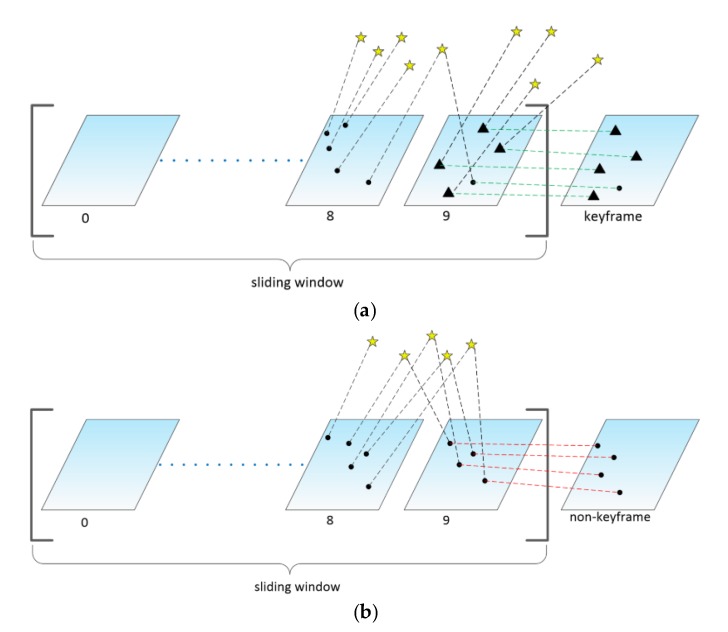
The top chart (**a**) shows how to track a keyframe. The black triangle represents the newly extracted feature points on the 10th frame (last frame) of the sliding window, while the green dotted line represents tracking them in the keyframe by KLT sparse optical flow algorithm. The bottom chart (**b**) shows how to track a non-keyframe. The red dotted line indicates that the feature points on the 10th frame of the sliding window are tracked on the non-keyframe by the direct method.

If the current frame is treated as a keyframe, we first use the fast feature detector [28] to add new feature points in the last frame in the sliding window and then use the KLT sparse optical flow algorithm to track them in the current frame (Figure 3). At least 200 feature points will be maintained in each frame. Since there is no need to calculate the feature point descriptor, the optical flow method can save more time. In addition, we also use RANSAC [29] with the fundamental matrix model to eliminate outliers generated during tracking.

### 4.3. Non-Keyframes Tracking

**Figure 4:**
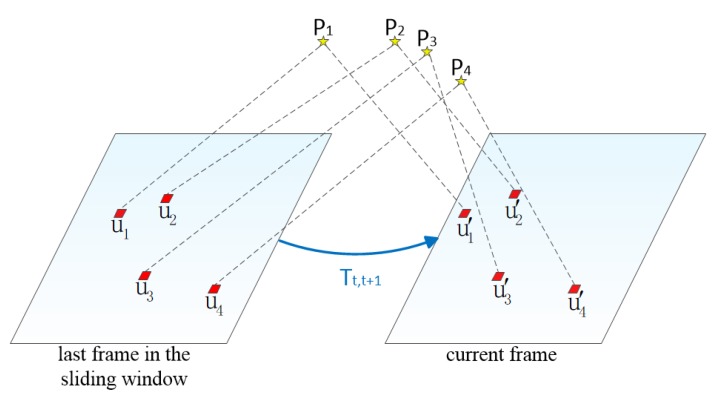
Adjusting the relative pose $T_{t,t+1}$ between the current frame and last frame in the sliding window means moving the re-projection position $u^{\text{′}}$ of feature points on the current frame.

If the current frame is considered a non-keyframe, we use direct image alignment to estimate the relative pose $T_{t,t+1}$ between the current frame and the last frame in the sliding window. The initial value of the relative pose can be obtained directly by IMU pre-integration. The feature points observed in the last frame are projected into the current frame according to the estimated pose $T_{t,t+1}$. Due to the hypothesis of photometric invariance, if the same feature point is observed by two adjacent frames, the photometric values of the projection points on the two adjacent frames are equal (Figure 4). Therefore, we can optimize the relative pose $T_{t,t+1}$ by minimizing the photometric error between image blocks (4 × 4 pixels):

(26) $$T_{t,t+1}=\arg\underset{T}{\min}\iint_{R}^{\;}\rho \left[\delta I\left(T,u\right)\right]du$$

where $\rho \left[\cdot \right]=\frac{1}{2}{\left\|\cdot \right\|}^{2}$, u is the position of the feature point of the last frame in the sliding window. R is the image region where the depth is known in the last frame, and the back-projected points are visible in the current frame domain.

The photometric error δI is:

(27) $$\delta I\left(T_{t,t+1},u\right)=I_{t+1}\left(\pi T_{t,t+1}\cdot \pi^{-1}\left(u,d_{p}\right)\right)-I_{t}\left(u\right)\;\;\;\;\forall u\in R$$

where $d_{p}$ is the depth of the feature point in the last frame in the sliding window. $I_{k}$ represents the intensity image in the k-th frame.

We use the inverse compositional formulation [30] of the photometric error, which can avoid unnecessary Jacobian derivation. The update step T(ξ) for the last frame in the sliding window is:

(28) $$\delta I\left(\xi ,u\right)=I_{t+1}\left(\pi T_{t,t+1}\cdot \pi^{-1}\left(u,d_{p}\right)\right)-I_{t}\left(\pi \left(T\left(\xi \right)\cdot \pi^{-1}\left(u,d_{p}\right)\right)\right)\;\forall u\in R$$

We solve it in an iterative Gauss Newton method and update $T_{t,t+1}$ in the following way:

(29) $$T_{t,t+1}\leftarrow T_{t,t+1}\cdot T{\left(\xi \right)}^{-1}$$

**Figure 5:**
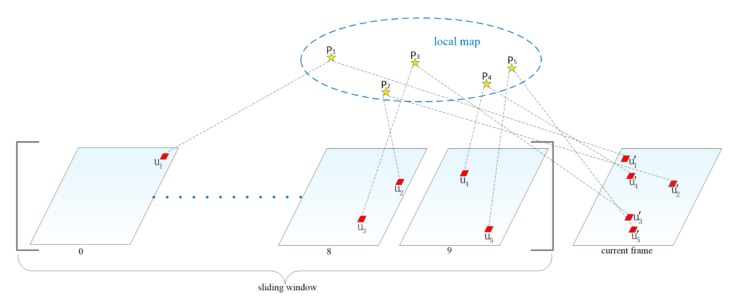
Adjust the position of the projection block $u_{i}^{\text{′}}$ on the current frame to minimize the photometric error of the projection block in the current frame and the reference frame in the sliding window.

After image alignment, we can get the optimized relative pose $T_{t,t+1}$ between the current frame and the last frame in the sliding window. We define all 3D points observed in all frames in the sliding window as the local map, and project the local map to the current frame to find the visible 3D points of the current frame. Due to the inaccuracy of the visible 3D point position and the camera pose, there will be errors in the projection position of the current frame. To make the projection position more accurate, the current frame needs to be aligned with the local map. The feature matching step optimizes the positions of all the projection points in the current frame by minimizing the photometric errors of the projection blocks (5 × 5 pixels) in the current frame and the reference frame (Figure 5):

(30) $$u_{i}^{\prime }=\arg\underset{u_{i}^{\prime }}{\min}\frac{1}{2}\|I_{t+1}\left(u_{i}^{\prime }\right)-A_{i}\cdot I_{i}{\left(u_{i}^{\;}\right)\|}^{2}\;\;\;\;\forall i$$

Solving the above formula in an iterative Gauss Newton method, we can get the update of the projection block position. The reference frame is usually far away from the current frame, so we apply an affine warping $A_{i}$ to the reference patch. 

Through image alignment and feature matching, we get the implicit results of direct motion estimation — feature correspondence with sub-pixel accuracy. Note that when tracking non-keyframes with the direct method, no new feature points are extracted. In the back-end optimization, we will combine IMU residual, visual re-projection error, prior information, and re-localization information to optimize the camera pose and 3D point position again.

## 5. Sliding Window-based Tightly-coupled Optimization Framework 

After tracking non-keyframes and keyframes, we proceed with a sliding window-based tightly-coupled optimization framework for high accuracy and robust state estimation. In the optimization framework, we combined IMU residual, visual re-projection error, prior information, and re-localization information to optimize the camera pose and 3D point position again.

### 5.1. Formulation

The state variables to be estimated by SD-VIS are defined as:

(31) $$X=\left[\begin{array}{ccc} X_{0} & X_{1} & \begin{array}{ccc} \ldots & X_{n} & X_{C}^{B} \end{array} \end{array}\right]\;\;\;\;\;\;{\;X}_{k}=\left[\begin{array}{ccc} P_{B_{k}}^{W} & V_{B_{k}}^{W} & \begin{array}{ccc} q_{B_{k}}^{W} & b_{\alpha }^{B} & b_{\omega }^{B} \end{array} \end{array}\right]\;k\in \left[0,n\right]\;X_{C}^{B}=\left[\begin{array}{cc} P_{C}^{B} & q_{C}^{B} \end{array}\right]$$

where $X_{k}$ includes the translation, velocity, and rotation quaternions of the $k^{\mathrm{th}}$ IMU body coordinate system concerning the world coordinate system, as well as the bias of gyroscope and accelerometer. n represents the size of the sliding window. By minimizing the sum of IMU residuals, visual re-projection errors, prior information, and relocation information in the sliding window, we can obtain a robust and accurate semi-direct visual-inertial SLAM system:

(32)  X min{∑​‖rB(Z˜Bk+1Bk,X)‖PBk+1Bk2+∑​‖rC(Z˜FCj,X)‖PFCj2+‖rp−HpX‖2+∑​‖rC(Z˜FCL,X)‖PFCL2}

where ${\;r}_{B}\left({\tilde{Z}}_{B_{k+1}}^{B_{k}},X\right)$, $r_{C}\left({\tilde{Z}}_{F}^{C_{j}},X\right)$, $\left\{r_{p},H_{p}\right\}$ and $r_{C}\left({\tilde{Z}}_{F}^{C_{L}},X\right)$ are IMU residuals, visual re-projection errors, prior information and re-localization information respectively.

### 5.2. IMU Residuals

According to the formulas (4)–(6) in Section 3.2, we can get the IMU measurement residual:

(33) $$r_{B}\left({\tilde{Z}}_{B_{j}}^{B_{i}},X\right)=\left[\begin{array}{c} {\delta \theta }_{B_{j}}^{B_{i}}\\ {\delta \beta }_{B_{j}}^{B_{i}}\\ {\delta \gamma }_{B_{j}}^{B_{i}}\\ {\delta b}_{\alpha }^{B}\\ {\delta b}_{\omega }^{B} \end{array}\right]={\left[\begin{array}{c} R_{W}^{B_{i}}\left(P_{B_{j}}^{W}-P_{B_{i}}^{W}-V_{B_{i}}^{W}\Delta t+\frac{1}{2}g^{W}{\Delta t}^{2}\right)-{\tilde{\theta }}_{B_{j}}^{B_{i}}\\ R_{W}^{B_{i}}\left(V_{B_{j}}^{W}-V_{B_{i}}^{W}+g^{W}{\Delta t}^{\;}\right)-{\tilde{\beta }}_{B_{j}}^{B_{i}}\\ 2{\left[q_{B_{i}}^{W}{}^{-1}⊗q_{B_{j}}^{W}⊗{\left({\tilde{\gamma }}_{B_{j}}^{B_{i}}\right)}^{-1}\right]}_{\mathrm{xyz}}\\ b_{\alpha }^{B_{i}}-b_{\alpha }^{B_{j}}\\ b_{\omega }^{B_{i}}-b_{\omega }^{B_{j}} \end{array}\right]}_{15*1}$$

where ${\left[\cdot \right]}_{\mathrm{xyz}}$ represents the real part of the quaternion. ${\left[{\tilde{\theta }}_{B_{j}}^{B_{i}},{\tilde{\beta }}_{B_{j}}^{B_{i}},{\tilde{\gamma }}_{B_{j}}^{B_{i}}\right]}^{T}$ are the IMU pre-integration between two adjacent keyframes $B_{i}$ and $B_{j}$.

### 5.3. Visual Re-Projection Errors

When the feature point $F_{1}$ is first observed in the $i^{\mathrm{th}}$ image, the visual re-projection error in the $j^{\mathrm{th}}$ image can be defined by the pinhole camera model as:

(34) $$r_{C}\left({\tilde{Z}}_{F_{1}}^{C_{j}},X\right)=\pi^{-1}(\left[\begin{array}{c} {\tilde{u}}_{F_{1}}^{C_{i}}\\ {\tilde{v}}_{F_{1}}^{C_{i}} \end{array}\right]-R_{B}^{C}(R_{W}^{B_{j}}(R_{B_{i}}^{W}(R_{C}^{B}\pi^{-1}\left[\begin{array}{c} {\tilde{u}}_{F_{1}}^{C_{j}}\\ {\tilde{v}}_{F_{1}}^{C_{j}} \end{array}\right]+P_{C}^{B})+P_{B_{i}}^{W}-P_{B_{j}}^{W})-P_{C}^{B})$$

where $\left[{\tilde{u}}_{F_{1}}^{C_{i}},{\tilde{v}}_{F_{1}}^{C_{i}}\right]$ and $\left[{\tilde{u}}_{F_{1}}^{C_{j}},{\tilde{v}}_{F_{1}}^{C_{j}}\right]$ represent the coordinates of the pixels projected from the feature point $F_{1}$ to the $i^{\mathrm{th}}$ and $j^{\mathrm{th}}$ frame image, respectively. $\pi^{-1}$ is the back-projection function of the pinhole camera model.

### 5.4. Marginalization Strategy

**Figure 6:**
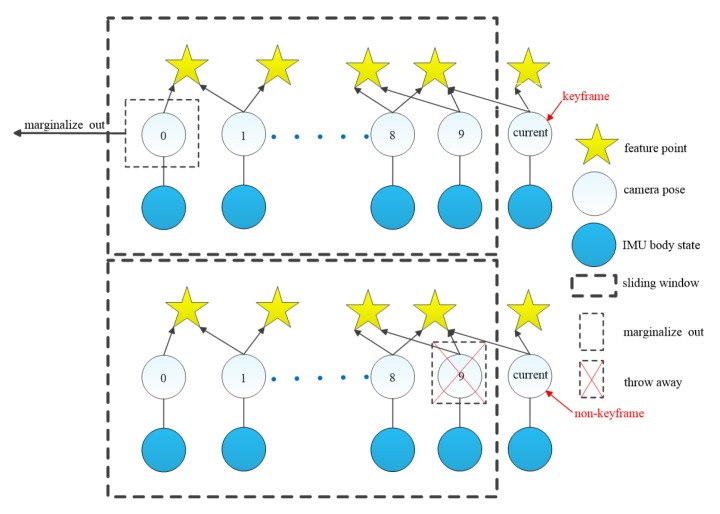
Marginalization strategy of SD-VIS.

In order to limit the computational complexity of SD-VIS, the back-end adopts a sliding window-based tightly-coupled optimization framework, so we use the marginalization strategy [31] to make the correct operation of sliding windows. As shown in Figure 6, if the current frame is determined as a keyframe, the frame will remain in the sliding window, and the oldest frame is marginalized out When the oldest frame is marginalized, the feature points that can only be observed by the oldest frame will be discarded directly, and other visual and inertial measurements associated with the frame will be removed from the sliding window by Schur complement. The new prior information constructed by Schur complement will be added to the existing prior information. If the current frame is not a keyframe, the last frame in the sliding window will be marginalized, and all visual measurements related to that frame will be removed directly from the sliding window.

### 5.5. Re-Localization

Due to the global 3D position and yaw angle are unobservable, there will be inevitable accumulative errors in the vision-inertial SLAM system. To eliminate the accumulated error, we introduce the re-localization module. After the keyframe is traced successfully, it can be judged whether the SLAM system has been here before by loop closure detection. We utilize DBoW2, a state-of-the-art bag-of-word place recognition approach, for loop closure detection. When a loop is detected, the re-localization module can effectively align the current sliding window, thus eliminating the accumulated error. For a detailed description of re-location, readers may refer to [18].

## 6. Experiment

We evaluate the accuracy, robustness, and real-time performance of SD-VIS on the EuRoC dataset [32]. The SD-VIS method is compared with the state-of-the-art vision SLAM methods, such as VINS-mono [18] and VINS-Fusion [20]. In Section 6.1, the accuracy and robustness of SD-VIS are evaluated, and the experimental results show that the accuracy and robustness of the proposed method reach the same level as the state-of-the-art method. Section 6.2 evaluates real-time performance. The experimental results show that the proposed method achieves a good balance between accuracy and real-time performance. Section 6.3 evaluates the loop closure detection capability and verifies the overall feasibility of the SLAM system.

### 6.1. Accuracy and Robustness Evaluate

In the experiments on the EuRoC dataset, we adopt the open source tool EVO [33] to evaluate the performance of SD-VIS. By comparing the estimated value with the actual value, we calculate the absolute pose error (APE) as an index of the evaluation algorithm [34]. Table 1 shows the root mean square error (RMSE) of the translation on the EuRoC dataset. For fairness, the following algorithms do not use the loop closure detection module.

**Figure 7:**
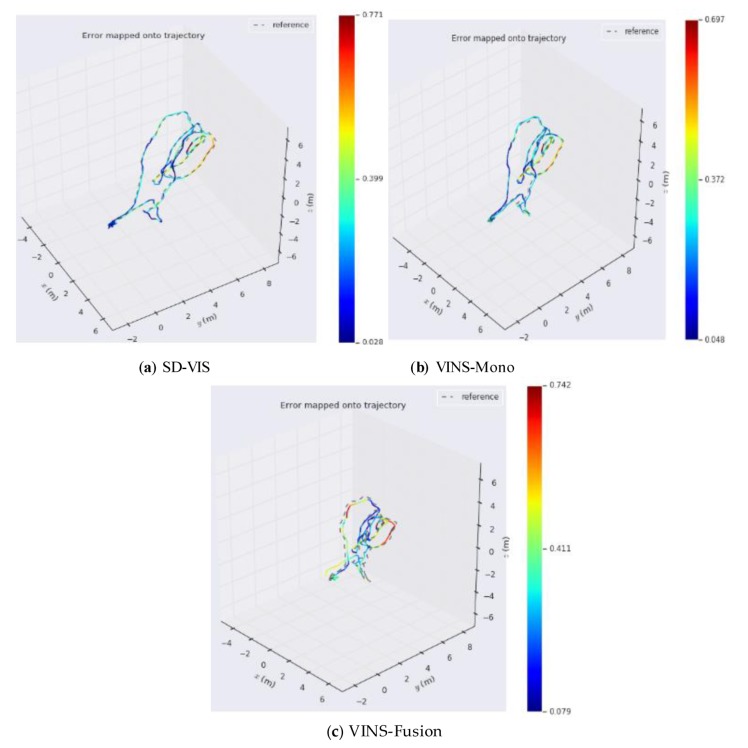
The trajectory heat map estimated by SD-VIS, VINS-Mono, and VINS-Fusion in MH_01_easy.

**Figure 8:**
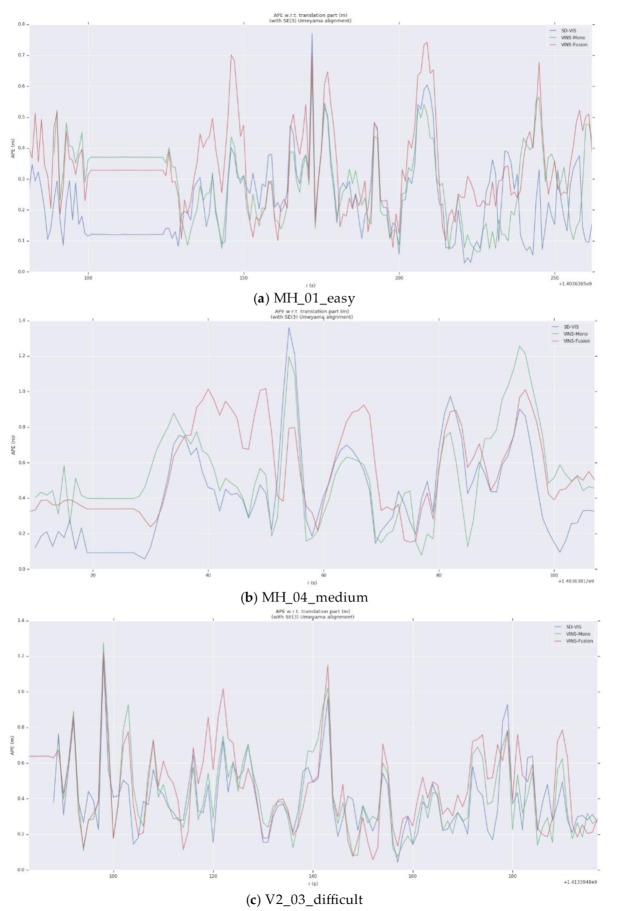
The change of translation absolute pose error with time in MH_01_easy, MH_04_medium and V2_03_difficult. Blue, green, and red represent SD-VIS, VINS-Mono, and VINS-Fusion respectively

As can be seen from Table 1, in terms of accuracy, SD-VIS and VINS-Mono and VINS-Fusion are at the same level. The accuracy of SD-VIS is slightly lower than that of VINS-Mono when moving at low and medium speed in the environment with abundant feature points (such as MH_01_easy and MH_03_medium). This is due to the susceptibility to various illumination changes when tracking non-keyframes using the direct method. We have observed that some datasets exhibit strong exposure changes between images and, therefore, the tracking effect of the direct method is reduced in these cases. In addition, in order to further improve the real-time performance of the algorithm, we only extract new feature points in keyframes, which results in that the number of feature points in non-keyframes will be less than keyframes, which will also bring some negative effects. Although the accuracy performance is not significantly better than the traditional method, it has achieved the same level of accuracy as VINS-Mono while greatly improving real-time performance. Therefore, our algorithm is very suitable for small-sized unmanned platform with limited computing resources. The accuracy of SD-VIS is higher than that of VINS-Mono and VINS-Fusion when moving fast in the low-texture environment (such as V2_03_difficult). This is due to the excellent performance of the direct method in the low-texture environment. In addition, the keyframe selection strategy will tend to generate more keyframes during fast motion, which will also improve the accuracy performance of the algorithm. Figure 7 shows more intuitively the trajectory heat map estimated by SD-VIS, VINS-Mono, and VINS-Fusion in MH_01_easy. Figure 8 shows the change of translation absolute pose error with time in MH_01_easy, MH_04_medium, and V2_03_difficult. Through Figure 7 and Figure 8, we came to the conclusion that the accuracy and robustness of our algorithm have reached the level of the state-of-the-art algorithm. Especially in the initialization procedure and low-texture environment, our algorithm performs better.

### 6.2. Real-Time Performance Evaluate

In this section, we evaluate the real-time performance of SD-VIS. We compared the average time required to track an image (Table 2).

As can be seen from Table 2, the image tracking of ORB-SLAM2 [11] uses the feature-based method to extract and match the ORB features of each frame, which takes a long time. However, VINS-Mono uses the optical flow method to track FAST features, which saves the calculation of feature descriptors, so the time consumption is less than ORB-SLAM2. VINS-Fusion is a stereo visual-inertial fusion SLAM algorithm, and image tracking also takes a long time. In SD-VIS, non-keyframes are used for fast-tracking and localization by direct method, and keyframes are tracked by feature-based method and used for back-end optimization and loop closure detection. This algorithm saves a lot of time and minimizes the average time of SD-VIS tracking images. Due to the keyframe selection strategy, in some low-speed motion scenes (such as MH_01_easy and V1_01_easy), the number of keyframes will be less, and the time to track a frame of the image will be reduced. In some fast-moving scenes (such as V1_02_medium and V2_03_difficult), as the number of keyframes increases, the time required to track an image will be increased.

In summary, the reason why we can obtain good real-time performance is due to the use of KLT sparse optical flow algorithm when tracking keyframes, which eliminates the calculation of descriptors and feature matching. In addition, for non-keyframes, only the direct method is used to track existing feature points, and new feature points are not extracted. Due to the close distance between two adjacent non-keyframes, the direct method of image alignment and feature matching can quickly converge.

**Figure 9:**
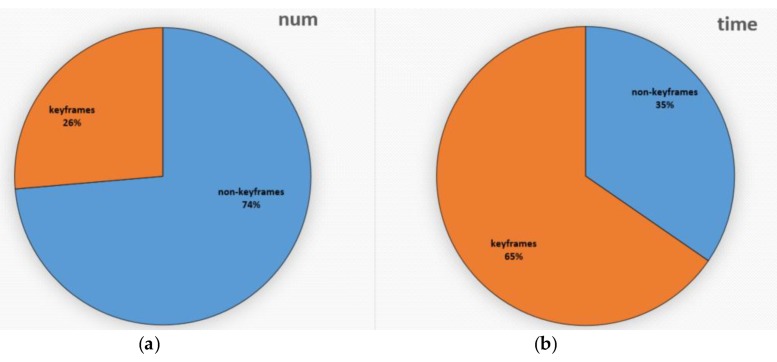
The left picture (**a**) shows a comparison of the number of keyframes and non-keyframes. The right picture (**b**) shows the comparison of time consumption between tracking keyframes and non-keyframes

Compared with the feature-based method, we use the direct method to track non-keyframes and accelerate the algorithm without reducing the accuracy and robustness. As shown in Figure 9, in MH_02_easy, 26% of the frames are determined to be keyframes, while 74% of the frames are determined to be non-keyframes. The time consumption of tracking keyframes is 65%, while that of non-keyframes are only 35%. Combined with Section 6.1, we can conclude that compared with the state-of-the-art SLAM systems, we can achieve a better balance between quickness and exactness.

### 6.3. Loop Closure Detection Evaluate

**Figure 10:**
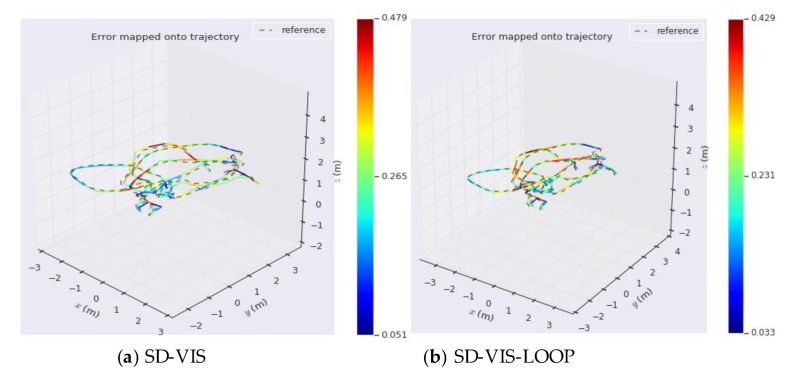
The trajectory heat map estimated by SD-VIS and SD-VIS-LOOP in V1_01_easy.

**Figure 11:**
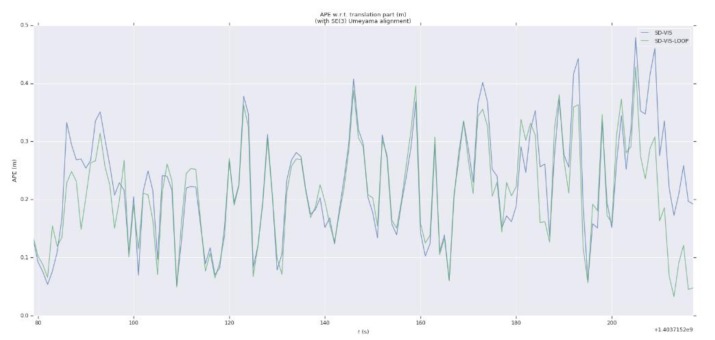
The change of translation absolute pose error with time in V1_01_easy. Blue and green represent SD-VIS and SD-VIS-LOOP respectively.

Finally, in order to verify the integrity and feasibility of the proposed algorithm, we evaluate the loop closure detection capability of SD-VIS. As can be seen from Figure 10 and Figure 11, the accuracy of SD-VIS with loop detection is improved obviously. Compared with the direct method, SD-VIS exhibits the function of loop closure detection and solves the problem of drift in long-term operation.

## 7. Conclusions

We present SD-VIS, a novel fast and accurate semi-direct visual-inertial SLAM framework, which combines the exactness of feature-based method and quickness of direct method. Compared with the state-of-the-art feature-based method, we use the direct method to track non-keyframes and accelerate the algorithm without reducing the accuracy and robustness. Compared with the direct method, SD-VIS exhibits the function of loop closure detection and solves the problem of drift in long-term operation. We get a better balance between accuracy and speed, so the algorithm is more suitable for the platform with limited computing resources. In the future, we will extend the algorithm to support more types of multi-sensor fusion to increase its robustness in complex environments.

## Figures and Tables

**Table 1:** The root mean square error (RMSE) results of the translation (m).

Dataset	VINS-Mono	VINS-Fusion	SD-VIS
MH_01_easy	**0.254651**	0.364247	0.260793
MH_02_easy	**0.263258**	0.339122	0.289663
MH_03_medium	0.547901	**0.483257**	0.577422
MH_04_medium	0.590191	0.614950	**0.497288**
MH_05_difficult	**0.512011**	0.524107	0.512458
V1_01_easy	**0.217083**	0.247467	0.245990
V1_02_medium	0.492645	**0.434756**	0.502134
V1_03_difficult	0.361521	**0.345895**	0.388959
V2_01_easy	**0.170790**	0.177467	0.202474
V2_02_medium	0.424259	**0.370081**	0.454785
V2_03_difficult	0.475561	0.521573	**0.444759**

**Table 2:** Average time (ms) spent tracking an image.

Dataset	ORB-SLAM2	VINS-Mono	VINS-Fusion	SD-VIS
MH_01_easy	37.82	17.67	38.91	**6.72**
MH_02_easy	35.31	13.60	45.86	**6.58**
MH_03_medium	34.20	12.28	44.15	**7.87**
MH_04_medium	30.35	12.92	42.54	**6.93**
MH_05_difficult	30.43	12.77	42.65	**6.91**
V1_01_easy	37.15	13.38	45.87	**7.14**
V1_02_medium	28.46	13.89	45.36	**10.96**
V1_03_difficult	×	13.50	44.40	**7.81**
V2_01_easy	33.01	15.30	49.10	**6.28**
V2_02_medium	31.13	13.04	45.56	**10.22**
V2_03_difficult	×	17.90	45.07	**12.38**
